# *Boechera* microsatellite website: an online portal for species identification and determination of hybrid parentage

**DOI:** 10.1093/database/baw169

**Published:** 2017-02-27

**Authors:** Fay-Wei Li, Catherine A. Rushworth, James B. Beck, Michael D. Windham

**Affiliations:** 1 Department of Biology, Duke University, Durham, NC 27708, USA; 2 University and Jepson Herbaria and Department of Integrative Biology, University of California, Berkeley, CA 94720, USA; 3 Department of Biological Sciences, Wichita State University, Wichita, KS 67260, USA; 4 Botanical Research Institute of Texas, 1700 University Drive, Fort Worth, TX 76107, USA

## Abstract

*Boechera* (Brassicaceae) has many features to recommend it as a model genus for ecological and evolutionary research, including species richness, ecological diversity, experimental tractability and close phylogenetic proximity to *Arabidopsis*. However, efforts to realize the full potential of this model system have been thwarted by the frequent inability of researchers to identify their samples and place them in a broader evolutionary context. Here we present the *Boechera* Microsatellite Website (BMW), a portal that archives over 55 000 microsatellite allele calls from 4471 specimens (including 133 nomenclatural types). The portal includes analytical tools that utilize data from 15 microsatellite loci as a highly effective DNA barcoding system. The BMW facilitates the accurate identification of *Boechera* samples and the investigation of reticulate evolution among the ±83 sexual diploid taxa in the genus, thereby greatly enhancing *Boechera*’s potential as a model system.

**Database URL:** http://sites.biology.duke.edu/windhamlab/

## Introduction

Among the close relatives of *Arabidopsis* Heynh., the genus *Boechera* Á. Löve & D. Löve (Brassicaceae) is notable for its species richness, ecological diversity and experimental tractability, features that have contributed to its emergence as a model genetic system for studies of evolutionary biology (1). However, efforts to fully leverage *Boechera* as a model system have been hampered by the taxonomic complexity of the genus and resultant misidentifications of materials used in various research studies. The taxonomic uncertainty in *Boechera* arises from a “perfect storm” of hybridization, polyploidy, and apomixis, all of which are rampant in the genus (2–7). Through these processes, the already substantial diversity of sexual diploid *Boechera* taxa (±83) has given rise to hundreds of additional taxa involving diverse combinations of at least 64 of these known sexual genomes (see 8–14).

In groups prone to such hybridization, determining which taxa represent the products of divergent evolution (i.e. sexual diploids) and which are the result of reticulate evolution is a prerequisite to developing a useable taxonomy (15–18). However, sexual diploids can be very difficult to detect morphologically in the apomictic hybrid milieu (14), and traditional molecular tools for species identification and hybrid diagnosis have proven of little use in *Boechera* due to widespread allele-sharing across species (19–21) and possible plastid capture (21). To address this problem, we have developed a 15-locus nuclear microsatellite ‘barcode’ that distinguishes all known sexual diploids and simultaneously provides a framework for determining the genomic make-up of hybrid individuals. These short genetic markers offer another advantage, in that they can be reliably amplified from herbarium specimens of *Boechera* collected over the past 130 years. Here we present a database of 56 353 microsatellite allele calls from 4471 specimens including 133 nomenclatural types. We also present novel tools for genotype-based species identification and determination of the genomic constitution of hybrid individuals.

## Materials and methods

### Microsatellite data

The database consists of microsatellite genotypes and basic locality data for 4471 *Boechera* specimens representing ca. 95% of all previously named taxa. Each population of every taxon included in the database is represented by voucher specimens deposited in one or more of the 36 herbaria supporting this effort. DNA extraction and multiplex microsatellite genotyping were performed using protocols outlined in Beck *et al.* (12). In addition to the 13-locus microsatellite set employed in that study, we incorporated two additional loci (BF19 and a3; Table 1). These loci are useful for sample identification but are excluded from heterozygosity calculations because these loci occasionally exhibit more than the expected number of alleles in plants of known ploidy. Extra alleles likely indicate amplification of multiple regions of the genome, and these loci are considered unreliable for heterozygosity estimates. The need for consistency in calling alleles is critical, and we have developed a set of locus-by-locus guidelines accessible through the *Boechera* Microsatellite Website (BMW) entry page by clicking on ‘About & Contact’.

**Table 1.** Information for the 15 microsatellite loci presented in the database

Marker	Primer sequences	Chr. no.	nt repeat	Number of alleles, N_a_	Original publication
**ICE3**	GACTAATCATCACCGACTCAGCCAC	5	CT	62	(22)
	ATTCTTCTTCACTTTTCTTGATCCCG				
**ICE14**	TCGAGGTGCTTTCTGAGGTT	2	GAT	17	(22)
	TACCTCACCCTTTTGACCCA				
**a1**	GTCTATTCGAGGACGCC	2	GAT	15	(23)
	AGGTTGGGTAGGTGAAG				
**a3**	AGCTTTGTTTGCAATGGAG	2	AG, AT	45	(23)
	GTGAGAATAATATTGACC				
**b6**	GCAAAAGATCTTCATGGGAC	1	CT, GT	62	(23)
	TGCCATTTCTTTCCCTAGTG				
**c8**	TTCCGGGTATCATTCCTAG	5	CTT	58	(23)
	GTTGTAAGTTCTTTCTCAG				
**e9**	GCGTATCTCGAATCACCTTTG	4	CT	56	(23)
	CTCCCCCTGAGTTTTTCAAG				
**BF3**	TTTTTAGACAGTAGTGGCTGTGAG	4	GA	58	(24)
	ACTTCGTTCCAGGCTCGTC				
**BF9**	AAACACATTCCCGTCAGCTC	3	GA	55	(24)
	TTGATTGAATCCTGCGTTTG				
**BF11**	TCCTCCATTGTAGAGCAGAGC	2	GA	27	(24)
	CCATTGCTTAAACCCTAAACC				
**BF15**	CAGCATCTCCTTTGGGTTTG	5	GA	48	(24)
	ACTTGCTCCTTTGCATGACC				
**BF18**	AACCTCCCAAGATTCGCTTC	1	CT	30	(24)
	TTCGCCATTGTTGTGATTTG				
**BF19**	ACCGCATTGGTGTTGTGTC	-	GA	63	(24)
	ATAACGGACGCGACCAAAG				
**BF20**	TTCTCGGGAAAGTAATGAGGAG	2	CT	47	(24)
	GCAAATCTGACCAATGCAAG				
**Bdru266**	TTTAATTTGTGCGTTTGATCC	-	AT	54	(24)
	CAAAATCGCAGAATGAGAGG				

Loci a3 and BF19 occasionally exhibit more than the expected number of alleles in plants of known ploidy and were therefore excluded from heterozygosity calculations. Null alleles were treated as missing data and are not included in the total number of alleles.

### Inferring reproductive modes

**Figure 1.**
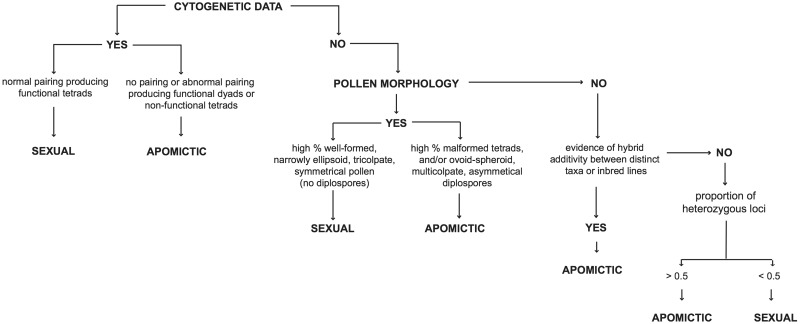
Summary of the steps involved in inferring reproductive mode in accessions of *Boechera*.

The two primary reproductive pathways in *Boechera* are sexuality (largely self-compatible and self-pollinating) and apomixis, the latter defined here as asexual seed production through either diplospory or apospory (25). The reproductive pathway of each accession in the database has been inferred based on a series of genetic and morphological assessments summarized in Figure 1. Foremost among these are analyses of meiotic chromosome pairing and segregation (14). Plants exhibiting normal pairing behavior resulting in the formation of spore tetrads are considered sexual; those with little or no pairing that produce malformed spores or functional diplospores in dyads are inferred to be apomictic. Including both published (summarized in 26) and unpublished chromosome studies, we have cytogenetic data for ∼5% of all accessions in the database. Inferring the reproductive mode of the remaining accessions requires some additional observations.

The differences in chromosome pairing cited above lead to easily observed disparities in pollen morphology between sexuals and apomicts. Following Windham and Al-Shehbaz (8) and Beck *et al.* (12), accessions with a high percentage of well-formed, narrowly ellipsoid, tricolpate and symmetrical pollen (and no diplosporous pollen) are inferred to be sexual; those with a preponderance of malformed (highly variable) and/or ovoid-spheroid, multicolpate and asymmetric pollen are classified as apomictic. Approximately 60% of *Boechera* herbarium specimens have flowers with sufficient pollen to ascertain reproductive mode. Assessment of reproductive mode in remaining specimens involves looking for evidence of stabilized hybridity in the available microsatellite data. Specimens exhibiting allelic additivity (involving either distinct taxa or inbred lines) at most loci are inferred to be apomicts, with the exception of a small number of sexual allotetraploids in the dataset. Accessions in which nearly all alleles are associated with a single taxon or inbred line are considered sexual.

### Inferring ploidy levels

Many previous studies have reported variation in ploidy between and within species of *Boechera* (see 23 and references therein). Voucher specimens for most published chromosome counts in the genus have been included in the database, as have the vouchers for several hundred unpublished counts (Windham *et al.*, in prep.). These data reveal a very strong association between the chromosome number documented for each individual and the maximum number of alleles observed at 13 of the 15 microsatellite loci analyzed (excluding loci a3 and BF19; Table 1). Cytogenetically known diploids in *Boechera* range from completely homozygous to having two alleles at 12 of these 13 putatively single-copy loci, whereas documented apomictic triploid and apomictic tetraploid individuals exhibit at least two loci with three or four alleles, respectively. These observations provide a pathway for inferring the ploidy of accessions that have not been studied cytogenetically but have appropriate microsatellite data. These inferences are based on the maximum number of alleles at two or more loci, a conservative threshold adopted to prevent the inflation of inferred ploidy based on a single aberrant locus or allele call. For example, a specimen would be called triploid if two or more of the loci used to assess heterozygosity (Table 1) exhibited three alleles. Plants that are homozygous at all loci are, by default, diploid. Although this approach works well for distinguishing diploids from triploids and apomictic tetraploids, sexual tetraploids are less tractable. The database includes 56 sexual tetraploid accessions representing two taxa: *holmgrenii* × *lemmonii* and *laevigata* × *stricta*. These hybrid species exhibit disomic inheritance (fixed heterozygosity) and rarely show more than two alleles per locus, as is common in sexual allotetraploids derived from relatively divergent species (26). Thus they are largely indistinguishable from highly heterozygous (apomictic) diploids based on the maximum number of alleles per locus. However, sexual tetraploids are rarely encountered in *Boechera* (27, 28), and they are readily separated from apomictic diploids by other criteria, including pollen morphology and chromosome number (Fig. 1).

**Figure 2.**
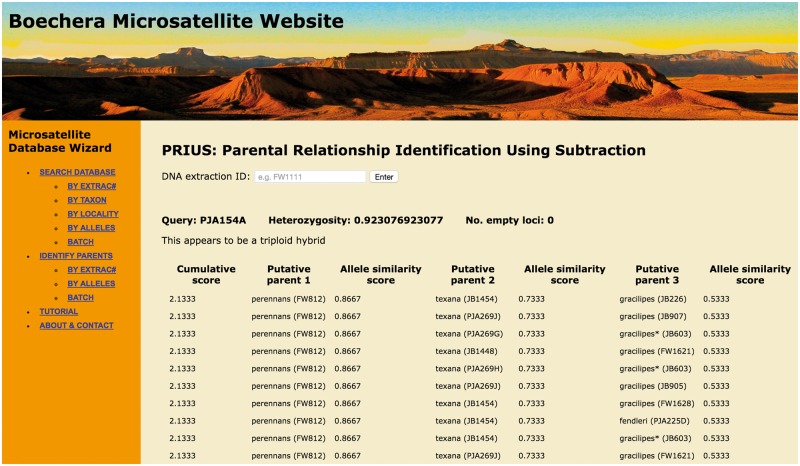
Screen shot of entry page for the BMW listing basic functions (left banner) and showing the result of a PRIUS search on Extrac# PJA154a.

### Taxonomic coverage

In the database as well as this article, known or inferred sexual diploid samples are designated by single epithets (e.g. ‘gracilipes’), most validly published in *Boechera* but with a few still pending. Inferred apomicts (diploid, triploid or tetraploid) and the few sexual tetraploids are identified by the appropriate number and combination of epithets, each separated by the hybrid symbol ‘×’ (e.g. ‘gracilipes × perennans × texana’). After removing 43 accessions that lack data at eight or more loci, the remaining 4428 accessions included 2594 presumed or known sexual individuals and 1834 likely apomicts (Table 2). These represent 485 unique entities (sexual diploid taxa and their hybrid derivatives), a phenomenal amount of diversification given the relatively short evolutionary history of the group (29). A full list of sexual diploid taxa and the current sampling of each can be accessed by clicking on ‘By Taxon’ on the entry page of the website. As of this publication, all known sexual diploid taxa except the critically endangered *B. perstellata* are represented in the database, with an average of 31 samples each. Several previously recognized rare endemics (e.g. *Boecherahoffmannii*, *Boecheraserotina*, *Boecherayorkii*) remain poorly sampled, as do many of the newly discovered sexual diploids (e.g. *‘roguensis’*, *‘tahoensis’*, *‘wallowaensis’*). In terms of geography, the region with the greatest taxonomic complexity (i.e. western North America) is generally well sampled. However, the various taxa confined to eastern North America and eastern Asia are currently under-sampled. Our ongoing research efforts are focused on filling these gaps, and the database will be continuously updated as new results become available.

**Table 2.** Summary of inferred ploidy levels and reproductive modes in 4428 *Boechera* database accessions after removing 43 accessions with >50% missing data

	Diploid	Triploid	Tetraploid	Total
**Sexual**	2538	0	56	2594
**Apomictic**	863	959	12	1834
**Total**	3401	959	68	4428

### Database structure

In order to facilitate the broadest usage of these data, the database has been made openly accessible through a web portal (http://sites.biology.duke.edu/windhamlab/), constructed using custom Python and HTML scripts, and connected by the Common Gateway Interface. The core is a Python library for interacting with the database (scripts available at https://bitbucket.org/fayweili/boecheradatabase.git). Users can search and retrieve basic metadata using the Extrac# (a unique ID number annotated on every voucher specimen), taxon name, or locality (Fig. 2). Searches based on taxon name can be limited to sexual diploids (i.e. just the epithet entered) or expanded to include all hybrids containing that genome. The output of a taxon query can be used to produce working state and county level distribution lists free of the misidentifications common in other on-line resources. The search by locality function allows investigators to quickly assemble checklists of confirmed *Boechera* identifications for any state, province, or county. This can improve the efficiency of morphology-based identification by reducing the diversity of taxa and the number of dichotomous key couplets that need to be negotiated. To further facilitate this process, exemplar specimens of each sexual diploid taxon as well as all documented hybrid combinations are being imaged and will be available via the portal. In addition to the basic search and display functions, we also include two novel algorithms: ‘Taxon Enquiry based on Similarity of Loci and Alleles’ (TESLA) for sample identification, and ‘Parental Relationship Identification Using Subtraction’ (PRIUS) for inferring the genomic constitution of hybrid individuals (see below). Although many microsatellite databases have been developed for plants, the majority of them focus on crop plants with the main purpose to assist breeding and genotyping marker selection (e.g. 30, 31). To our knowledge, the only comparable database for biodiversity research and species identification is the Olive Genetic Diversity Database (32).

### Taxon enquiry based on similarity of loci and alleles (TESLA)

Taxonomic identification of *Boechera* specimens is notoriously challenging, even for specialists (11). With about ±83 sexual diploid taxa that hybridize whenever they come into contact to produce true-breeding apomictic diploids, triploids and tetraploids, it is impossible to identify many accessions based on gross morphology alone (14). To address this problem, we have developed TESLA, a microsatellite-based identification method. Once a user enters allelic information for their sample, TESLA compares this multi-locus genotype (MLG) to every accession in the database, calculating an Allele Similarity (AS) score between two accessions as follows:

$$AS\;=\frac{\left(No.\;shared\;alleles\right)\times (No.\;loci-No.\;loci\;without\;data)}{\left(Total\;allele\;count\;in\;query)\times (No.\;loci\right)}$$

The AS score represents the proportion of alleles that are shared relative to the query, scaled by the amount of missing data. The output of a TESLA query consists of the specimens with the 100 highest AS scores listed in descending order. In addition to researching samples already included in the database, user-generated MLGs can be entered individually (using the ‘by alleles’ function) or a query spreadsheet can be uploaded to the database in batch mode (Fig. 2).

Both the rank value and magnitude of the AS scores derived from TESLA are useful in identifying unknown *Boechera* accessions. For example, in queries involving 100 random sexual diploid samples of known identity, the highest AS scores coincided with the ‘correct’ identification 96% of the time. In an additional 3%, the highest scoring matches belonged to the same major clade as the expected taxon (clade assignments of all sexual diploid taxa can be accessed through ‘Search Database By Taxon’). These phylogenetically localized mismatches were attributable to limited sampling of the taxon involved, poor differentiation between the target taxon and its close relatives, and/or missing data. In the single instance in which the individual with the highest AS score belonged to an unrelated taxon, the analysis was impeded by a lack of data at the majority of loci. In terms of magnitude, the highest AS scores from TESLA searches resulting in correct identifications ranged from 1.000 to 0.375. Misidentifications that attribute a sample to the wrong clade became common below 0.500 and we therefore recommend this as the threshold for considering a TESLA result a hypothesis worthy of serious consideration.

### Parental relationship identification using subtraction (PRIUS)

*Boechera* hybrids are ubiquitous, often comprising >50% of plants randomly sampled from natural populations (M. D. Windham, personal observation). They are also hyper-diverse, drawing nearly every known sexual genome into the apomictic milieu (12, 13). Few extant analytical programs can determine the genomic constitution of hybrid individuals based on microsatellite data (33–35), and none of the available implementations are capable of handling the tri-genomic hybrids common in *Boechera*. To address this need, we have developed a program called ‘PRIUS’, available via the online database portal.

The basic workflow of PRIUS is as follows: (i) identify the most similar sexual diploid specimens in the database based on AS score, (ii) subtract from the hybrid’s MLG the corresponding alleles from each top-scoring specimen and (iii) repeat the previous steps using the subtracted genotype for one or two more iterations (see below). The end result is a list of diploid specimen combinations that best explain the hybrid’s MLG. The corresponding species of these diploid specimens then form a working hypothesis of the putative parental species.

The proper functioning of PRIUS depends on the ability to distinguish between sexual diploids (the pool from which potential parents are drawn) and apomicts (generally the products of hybridization; see 12, 36). Fortunately, this information resides within the microsatellite data themselves. Based on a broad taxonomic sampling of 13 microsatellite loci in 1393 *Boechera* specimens, Beck *et al.* (12) demonstrated clear bimodality in the number of heterozygous loci, a metric that was strongly associated with inferred breeding system. In this study, the mean heterozygosity of diploid plants with predominantly sexual pollen was 0.232 (3.02/13), whereas the mean for diploid plants with predominantly apomictic pollen was 0.723 (9.4/13). Only one sexual diploid taxon (*parishii*) included multiple samples in which heterozygosity exceeded 0.5, and only 6 of 82 apomictic diploid taxa exhibited heterozygosity <0.5 (12). Based on this empirical evidence, we set the PRIUS heterozygosity threshold at 0.5, thus creating essentially non-overlapping pools of potential parents (sexual diploids) and apomictic (or sexual tetraploid) hybrids subject to a PRIUS query.

To begin the PRIUS process, the program infers the ploidy of the query accession based on the maximum number of alleles at two or more of the 13 loci used to calculate heterozygosity (see Table 1). If the accession is determined to be triploid, PRIUS identifies the five sexual diploid specimens in the full dataset with the highest AS scores relative to the queried MLG. For each tri-allelic locus in the query accession, PRIUS independently subtracts from the MLG any matching alleles observed in each of the five sexual diploids, generating five new query MLGs. These roughly approximate apomictic diploid progenitors that could have hybridized with the subtracted genome to form the original triploid MLG. Each of the five subtracted MLGs is then queried in a second iteration, where PRIUS identifies the five sexual diploid specimens with the highest AS scores relative to each of the subtracted MLGs. For each di-allelic and tri-allelic locus in the five queried MLGs, the program subtracts any matching alleles from each of the five most similar sexual diploids. This step generates 25 (5^2^) new query MLGs that very roughly approximate a third sexual diploid genome that might have combined with the two subtracted genomes to form the original triploid MLG. A third iteration then identifies the five sexual diploid specimens with the highest AS scores relative to each of the 25 new subtracted MLGs. After three iterations, there will be 125 (5^3^) different specimen combinations, and PRIUS ranks them by cumulative AS scores. If a query accession is determined to be diploid (i.e. exhibits a maximum of two alleles at 12 of the 13 loci used to assess heterozygosity), PRIUS begins with the second iteration. As with TESLA, a user generated MLG can be entered individually (using the ‘by alleles’ function) or a query spreadsheet can be uploaded to the database in batch mode (Fig. 2).

PRIUS is not designed to directly address the parentage of the few tetraploid apomicts encountered (12/4428 specimens or 0.3% of the dataset) because a fourth round of subtraction produces a high percentage of spurious matches. PRIUS searches are also negatively affected by the presence of undetected null alleles in a queried accession. This is because the AS scores of appropriate sexual diploid accessions with null alleles are lowered, reducing their likelihood of being identified as possible parents. The effect can be significant, resulting in a bias against certain sexual diploid taxa (e.g. *retrofracta*) that are extensively involved in the formation of apomictic hybrids (11). This problem is not easily addressed, but we have instituted an approach that improves the success rate in both PRIUS and TESLA for many triploid accessions.

In sexual taxa, null alleles are hypothesized to exist in the homozygous state if both of the following conditions were met: (i) the remaining multiplexed loci from that individual amplified, and (ii) individuals in the same genotyping run representing other taxa that lack null alleles amplified. Because most *Boechera* triploids (even those arising within named species) combine divergent genomes with different alleles, we expect a given triploid sample to be tri-allelic or, at least, di-allelic at the majority of these highly variable loci (Table 1). A broad survey of the database suggests that the occurrence of apparently homozygous loci in such accessions is more often the result of undetected null alleles than the improbable incorporation of three identical alleles from three different parental genomes. Both PRIUS and TESLA therefore add a null allele (as ‘0’) to apparently homozygous loci in triploid queries. This protocol has no effect on the AS scores of accessions supporting the ‘three identical alleles’ hypothesis but increases the probability that sexual diploids with null alleles will be considered as possible parents.

It should be stressed that although PRIUS is a powerful tool for data exploration and generating hypotheses regarding the genomic constitution of hybrid *Boechera* individuals, the hypothesized hybrid origin should be evaluated with the widest array of data that can be brought to bear, including morphology, cytology, ecology, geography, genomics and reproductive biology (13).

## Results

The following examples demonstrate the functionality of TESLA (Table 3) and PRIUS (Fig. 2) for identifying hybrids. The sample queried (Extrac# PJA154a) was subject to a variety of analyses by Alexander *et al.* (13) and thus provides a good basis for comparing the outputs of these algorithms to other approaches used to identify taxa and propose hybrid parentages. Table 3 shows the result of a TESLA search on PJA154a, demonstrating that all of the most similar accessions in the database (with AS scores ranging from 1.0000 to 0.9143) have been identified as triploid hybrids incorporating genomes derived from *gracilipes*, *perennans* and *texana*. To facilitate presentation of this TESLA query, the full 15-locus dataset has been reduced to the eight loci that show allelic variability within this hybrid cluster. In addition, the standard output of TESLA (i.e. the 100 most similar accessions in the database) has been condensed by grouping hybrids with identical genotypes and assigning members of individual sexual diploid taxa with identical AS scores to a single row (the number of condensed accessions indicated parenthetically in the Extrac# column).

**Table 3.** Output from a TESLA query on Extrac# PJA154a

**AS_score**	**Extrac#**	**Taxon ID**	**State**	**County**	**ICE3**	**ICE3**	**ICE3**	**c8**	**c8**	**c8**	**ICE14**	**ICE14**	**BF3**	**BF3**	**BF3**	**b6**	**b6**	**b6**	**BF19**	**BF19**	**BF19**	**BF15**	**BF15**	**BF15**	**Bdru266**	**Bdru266**	**Bdru266**
	Query= PJA154a	gracilipes x perennans x texana	NM	Eddy	71	75	93	244	250		211	223	107	111	129	299	309	311	145	0		93	104	115	133	146	
1	PJA154b (3)	gracilipes x perennans x texana	NM	Eddy	71	75	93	244	250		211	223	107	111	129	299	309	311	145			93	104	115	133	146	
0.9722	PJA163h (6)	gracilipes x perennans x texana	NM	Doña Ana	71	75	93	244	250		211	223	107	111	129	299	309	311	145			93	104	115	133	154	
0.9722	PJA382z (2)	gracilipes x perennans x texana	NM	Torrance	71	75	93	244	250		211	223	107	111	129	299	309	311	145			93	104	115	133	150	
0.9722	PJA380a	gracilipes x perennans x texana	NM	Torrance	71	75	93	244	250		211	223	107	111	129	299	309	311	145			93	104	115	133	152	
0.9722	PJA385f	gracilipes x perennans x texana	NM	Otero	71	75	93	244	250		211	223	107	111	129	299	309	311	145			93	104	115	133	142	
0.9459	PJA380z	gracilipes x perennans x texana	NM	Torrance	71	75	93	244	250		211	223	107	111	129	299	309	311	143	145		93	104	115	133	152	
0.9444	PJA230a (2)	gracilipes x perennans x texana	NM	Doña Ana	71	75	93	244	250		211	223	107	111	129	299	307	311	145			93	104	115	133	150	
0.9444	PJA230c (2)	gracilipes x perennans x texana	NM	Doña Ana	71	75	93	244	250		211	223	107	111	129	299	307	311	145			93	104	115	133	152	
0.9211	JB1346 (2)	gracilipes x perennans x texana	NM	San Miguel	71	75	93	244	250		211	223	107	111	129	299	309	311	143	145		93	104	115	130	133	150
0.9189	PJA222e (2)	gracilipes x perennans x texana	NM	Doña Ana	71	75	93	244	250		211	223	107	111	127	299	309	311	145			93	104	115	130	133	150
0.9189	PJA385z (3)	gracilipes x perennans x texana	NM	Otero	71	75	93	244	248	250	211	223	107	111	129	299	309	311	145			93	104	115	131	148	
0.9167	JB1345	gracilipes x perennans x texana	NM	Socorro	71	75	93	244	250		211	223	111	127		299	309	311	145			93	104	115	133	136	152
0.9143	PJA395c (4)	gracilipes x perennans x texana	NM	Lincoln	71	75	77	244	250		211	223	107	111	129	299	309		145			93	104	115	131	150	
0.8667	FW812	perennans	AZ	Maricopa	75			244	258		223		111	155		307			145	153		93			141	150	
0.8667	FW483	gracilipes	AZ	Gila	71			250	252		223		105	107		317	321		147			104			125	133	
0.8	FW799 (4)	perennans	AZ	Gila	75			244			0		111	135		305	309		157	163		93			131	154	
0.7407	FW765	gracilipes x perennans x texana	NM	Doña Ana	71	75	93				223		107	111	127				145			93	104	115	130	133	
0.7333	PJA151h (14)	perennans	NM	Doña Ana	75			248	254		223		99	129		305	319		149			93			125	146	
0.7333	JB1454 (8)	texana	TX	Jeff Davis	73			232			211		97	123		313			0			101	115		127	146	
0.7143	JB595	gracilipes x perennans	AZ	Cochise	71	75		248	252		223		105	129		311	313		145	151	155	93	104		133	141	
0.6667	FW811 (30)	perennans	AZ	Maricopa	75			264			223		119			305			0			93			133		
0.6667	JB905 (6)	gracilipes	AZ	Coconino	71			258			223		99			321			145	151		104			0		
0.6667	JB183 (2)	spatifolia	CO	Fremont	75			242			223		101			311			143			99			133		
0.6667	JB1447	texana	TX	Brewster	73			232	240		211		107	115		313	319		0			101	137		137	144	
0.6538	JB648	gracilipes x perennans	AZ	Graham	73	75		252	262		223		111	119		309	315		145			93	104		156		
0.6111	JB1453	gracilipes x perennans x texana	TX	Culberson	73	75		232	242	248	211	223	107	113	125	299	309	315	147	151	155	93	104	137	146	148	

As indicated earlier, the top hits for a TESLA query on PJA154a are all specimens identified as *gracilipes* × *perennans* × *texana*. These comprise 13 different MLGs representing 30 individual samples (Table 3). In fact, all but two of the samples in the dataset with this hypothesized parentage are clustered at the top of this figure with AS scores ≥ 0.9143. One exception is Extrac# FW765 (Table 3; row 19), which is identical to PJA222e (row 12) except that it lacks data at two loci (c8 and b6) and has incomplete data at two others (ICE14 and Bdru266). The remaining exception to the tight clustering of samples identified as *gracilipes* × *perennans* × *texana* is Extrac# JB1453 (Table 3; last row), which has an AS score of 0.6111 reflecting divergent allele profiles at seven of the eight illustrated loci. We suspect that Extrac# JB1453 represents an independent hybrid origin of this triploid apomict, a hypothesis discussed in more detail below.

Random sampling of apomictic hybrids in the current dataset indicate that ca. 60% of TESLA searches correctly specify their genomic make-up, placing plants with identical parentage at the top of the results list. In the remaining 40% of cases in which highly similar hybrid genotypes have not yet been sampled, the probable parents typically are discernable among the sexual diploid taxa with high AS scores. Our query involving PJA154a (Table 3) can be used to illustrate this approach as well. Ignoring for the moment the hybrid origin suggested by rows 3–15, the next highest AS score (0.8667) is shared by individuals of *perennans* and *gracilipes*. A third sexual diploid taxon (*texana*) appears at an AS score of 0.7333. Repeated occurrences of these three species [as well as *gracilipes* × *perennans*, a possible apomictic diploid bridge discussed by Alexander *et al.* (13)] suggest the same hybrid parentage reflected in rows 3–15.

PRIUS provides an alternative, taxon-centric approach to assessing hybrid parentage and is especially informative in cases where TESLA fails to provide a clear result. Figure 2 presents a screen shot of the PRIUS query result for Extrac# PJA154a, the same individual used in the preceding TESLA search. The cumulative scores on the left serve the same function as the AS scores in TESLA. In the case of PJA154a, the highest cumulative score is 2.1333, which is represented by 92 rows (different combinations of individual accessions), with 10 presented in Figure 2, for clarity. In every case, putative parent 1 is an accession of *perennans* while putative parent 2 is *texana*. In 82/92 combinations (89.1%), an accession of *gracilipes* appears as putative parent 3. In another six cases (6.5%), *perennans* is repeated as the third parent and, in four others (4.4%), *fendleri* is identified as putative parent 3. All four of these sexual diploid taxa belong to the same major clade of *Boechera* (13, 21).

In Alexander *et al.* (13) Extrac# PJA154a is labeled PJA195a (the original collector’s number) and is identified as *Boechera porphyrea* (the correct binomial name for hybrids of this proposed parentage). In a phylogenetic analysis incorporating data from the nuclear *pistillata* locus, different clone sequences from this sample associated with *gracilipes*, *perennans* and *texana*, respectively (13). STRUCTURE analysis of the microsatellite data available at the time for this taxon revealed admixture proportions averaging 25% *gracilipes*, 37% *perennans* and 23% *texana*, with a lesser contribution attributed to *fendleri*. Thus, the results reported by Alexander *et al.* (13) are nearly identical to those obtained from our TESLA and PRIUS queries on PJA154a. Our new algorithms have two clear advantages, however. First, using TESLA and PRIUS requires no specialized taxonomic knowledge of the group. To conduct their analyses, both Alexander *et al.* (13) and Beck *et al.* (12) had to decide *a priori* which *Boechera* species were likely involved in the hybridization events they were trying to reconstruct. Second, TESLA and PRIUS results are obtained in seconds, as opposed to the time-consuming STRUCTURE algorithm.

## Discussion

In addition to providing microsatellite-based tools to facilitate the identification of *Boechera* specimens, the database also can be used to explore large-scale evolutionary patterns within the genus. Insights regarding the relationships among ploidy, heterozygosity and reproductive mode, and processes of hybrid formation and evolution are discussed below.

### Relationship between reproductive mode and ploidy

*Boechera* ploidy is non-randomly associated with reproductive mode (28). The clearest such association involves plants with three full sets of chromosomes (2*n* = 21) that have no chance of pairing normally in meiosis. The current dataset includes 959 triploid accessions (Table 2), all of which are inferred to be apomictic based on the stepwise analyses outlined in Figure 1. Whereas triploids represent 21.7% of the current dataset, tetraploids are rare (1.5%) and higher ploidy levels have not been observed. Based on cytogenetic and pollen data, the majority of tetraploid individuals are sexual; though 12 apomictic tetraploids (representing seven different genomic combinations) are present as well (Table 3). The two types of tetraploids are readily separated based on the maximum number of alleles per locus (two or rarely three in sexual tetraploids vs. four in apomicts). The remaining accessions (77.8%) are considered diploid based on the allele number criterion discussed earlier. Although ca. 75% of these appear to be sexual, our sampling was biased toward sexual individuals, and apomictic diploids are likely to be more prevalent in nature. This is a critical feature of the *Boechera* model system given the rarity of apomictic diploids among flowering plants (25).

### Relationship between reproductive mode and heterozygosity

**Figure 3.**
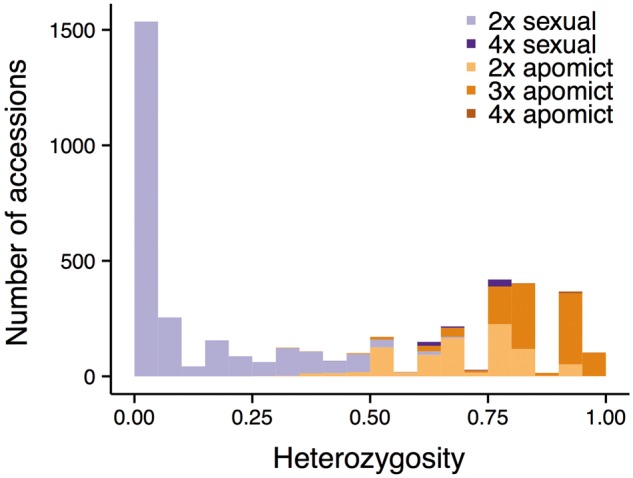
Histogram of heterozygosity for 4428 *Boechera* accessions included in this study, colored by reproductive mode (sexuals in purple and apomicts in orange) and ploidy level (darker with increasing ploidy).

As suggested by previous studies, apomicts exhibit higher levels of heterozygosity than sexually reproducing *Boechera* accessions (Fig. 3). This is not surprising given that the observed heterozygosity is largely the result of hybridization between different inbred genotypes, which may be a trigger for the development of apomixis (12, 37). Results from a Wilcoxon rank sum test of the current dataset indicate that apomict heterozygosity is significantly higher than that of sexuals (apomict mean 0.78 ± 0.14 SD, median 0.77 vs. sexual mean 0.11 ± 0.18 SD, median 0; Wilcoxon *W* = 4709860, *P* < 2.2e^−^^16^; Fig. 3). Although mean heterozygosity of triploid (0.85 ± 0.10 SD) and tetraploid apomicts (0.90 ± 0.07 SD) is higher than that of diploid apomicts (0.70 ± 0.13 SD), this result does not change qualitatively when comparing diploid only sexuals and apomicts.

The data presented in Figure 3 provide strong empirical support for using the 0.5 heterozygosity threshold implemented in PRIUS to separate potential parents (sexual diploids) from the non-segregating hybrids subject to analysis. Just 147 accessions (3.3% of the dataset) exhibit heterozygosity values higher or lower than expectations based on reproductive mode inferred via the pathway outlined in Figure 3. Unexpectedly high values are observed in 56 accessions of the two sexual tetraploid taxa (*holmgrenii* × *lemmonii* and *laevigata* × *stricta*) discussed earlier and 28 individuals of the sexual diploid taxon *parishii*. This species exhibits unusually high allelic diversity and possesses a suite of traits that suggest or enforce outcrossing. Unexpectedly low values are observed in a series of apomictic diploids that include: (i) a variety of hybrids between closely related sexual diploids that are insufficiently divergent to meet the 0.5 threshold when crossed, and (ii) a series of apomictic diploid hybrids that include *retrofracta* or other species with null alleles in their parentage.

### Hybrid formation and evolution

Earlier articles hint at the important role hybridization has played in the evolutionary diversification of *Boechera* (12, 13), but we clearly establish that hybridization is ubiquitous and largely unconstrained by phylogeny. Of the 485 taxa included in the current database, 400 are confirmed or inferred apomictic hybrids. Eighteen of these have arisen through crosses between divergent inbred lines within particular sexual diploid taxa; the remaining 382 are the products of hybridization between distinct taxa. Of the 83 sexual diploid taxa, 64 have been involved in at least one hybridization event and the 19 sexual diploid taxa not currently known to hybridize are those that rarely co-occur with other taxa and/or are currently under-sampled.

Hybridization has also occurred repeatedly among the same sexual diploid taxa, producing hybrid lineages with similar taxonomic constitutions but different parental alleles. The results for a TESLA query for Extrac# PJA154a, provide a good example (Table 3). The queried specimen, an apomictic triploid hybrid incorporating genomes derived from *gracilipes*, *perennans* and *texana* (13), belongs to a cluster of 13 very similar MLGs with AS scores ≥ 0.9143 and one outlier (Extrac# FW765, AS 0.7407) identical to one of these except for missing data. There is, however, a 14th MLG represented by a single sample (Table 3; Extrac# JB1453, row 28) with a much lower AS score of 0.6111. The lower score reflects the fact that there are allelic differences between JB1453 and the other 13 MLGs at nearly every locus analyzed. Given the lack of chromosome pairing and allelic segregation in these apomictic triploids, it is very likely that the apomictic triploid *gracilipes* × *perennans* × *texana* had at least two independent origins. A cursory survey of the data included in the BMW indicates that multiple origins of *Boechera* hybrid apomicts are the rule rather than the exception.

## Conclusion

The taxonomic complexity of *Boechera* has long stymied those investigating the diversity, biogeography, and evolutionary dynamics of the group (8, 11). Here we present a database containing information for 4428 specimens collected throughout the native range in North America, Greenland and Russia and encompassing >95% of all named taxa. A variety of search algorithms facilitate assigning the correct taxon name, ploidy, reproductive mode, and hybrid parentage to any *Boechera* specimen with appropriate microsatellite data. Two novel identification algorithms, TESLA and PRIUS, facilitate the identification of unknown *Boechera* specimens using simple, standardized methods. This database, publicly available and regularly updated, will provide valuable new tools for researchers interested in *Boechera* as a model genus.
